# A 2-transistor/1-resistor artificial synapse capable of communication and stochastic learning in neuromorphic systems

**DOI:** 10.3389/fnins.2014.00438

**Published:** 2015-01-15

**Authors:** Zhongqiang Wang, Stefano Ambrogio, Simone Balatti, Daniele Ielmini

**Affiliations:** Dipartimento di Elettronica, Informazione e Bioingegneria, Politecnico di Milano and IU.NETMilano, Italy

**Keywords:** neuromorphic circuits, spike timing dependent plasticity, neural network, memristor, pattern recognition, cognitive computing

## Abstract

Resistive (or memristive) switching devices based on metal oxides find applications in memory, logic and neuromorphic computing systems. Their small area, low power operation, and high functionality meet the challenges of brain-inspired computing aiming at achieving a huge density of active connections (synapses) with low operation power. This work presents a new artificial synapse scheme, consisting of a memristive switch connected to 2 transistors responsible for gating the communication and learning operations. Spike timing dependent plasticity (STDP) is achieved through appropriate shaping of the pre-synaptic and the post synaptic spikes. Experiments with integrated artificial synapses demonstrate STDP with stochastic behavior due to (i) the natural variability of set/reset processes in the nanoscale switch, and (ii) the different response of the switch to a given stimulus depending on the initial state. Experimental results are confirmed by model-based simulations of the memristive switching. Finally, system-level simulations of a 2-layer neural network and a simplified STDP model show random learning and recognition of patterns.

## Introduction

Brain-inspired computing is among the top challenges of the today's information and communication technology. The brain is capable of formidable tasks, such as learning, recognition of visual/auditory patterns, and adaptation in response to new information. To meet this grand challenge, a neuromorphic system should include a number of neurons and synapses similar to a biological human brain, featuring around 10^12^ neurons and 10^15^ synapses (Rajendran et al., 2013). Clearly, such a complex system can be realized only through advanced manufacturing techniques (e.g., 3D integration), and small circuit blocks for neurons and synapses. The latter, in particular, represents by far the largest area of the neuromorphic circuit due to the huge number of inter-neural connections, therefore scaling down the size and complexity of the artificial synapse is a key task in the design of a neuromorphic circuit.

To this purpose, nanoscale resistive switches, or memristors, have been proposed as novel artificial synapses in neuromorphic systems (Likharev et al., 2003; Snider, 2008; Jo et al., 2010). Memristors have the capability of an inherent analog tuning, combined with a 2-terminal structure and a scalable device area and power, therefore they display strong advantage with respect to silicon-based synapses, such as floating gate memories (Diorio et al., 1996) and static RAM (Indiveri et al., 2006). Different switch technologies have been proposed for artificial synapses, including phase change memories (Wright et al., 2011; Bichler et al., 2012; Kuzum et al., 2012), organic-based switches (Bichler et al., 2010), chalcogenide-based switches (Ohno et al., 2011; Suri et al., 2013) and oxide-based resistive switching memories (Seo et al., 2011; Yu et al., 2011, 2013; Park et al., 2012; Ambrogio et al., 2013). The latter approach provides analog switching, nonvolatile behavior, CMOS compatible materials, back-end process and scalable power consumption thanks to filamentary switching (Wong et al., 2012). A memristor naturally satisfies the requisites for electrically-tunable conductance, serving as a connection for communication between a pre-synaptic neuron (PRE) and a post-synaptic neuron (POST), and responsive to the individual spikes fed from both neurons. To achieve this multitask operation, a time-division multiplexing (TDM) approach was previously proposed, where neuron spikes obey a precise synchronous sequence for communication, long-term potentiation (LTP) and long-term depression (LTD) (Snider, 2008; Jo et al., 2010). The synchronous approach, however, may be too idealized with respect to the biological brain, where synapses are potentiated/depressed through asynchronous spike timing dependent plasticity (STDP) (Bi and Poo, 1998). Also, synchronous clocking may be practically difficult in the case of large neuromorphic systems (Zamarreño-Ramos et al., 2011). More recently, a fully asynchronous approach for communication/learning of neuromorphic synapses with leaky-integrate-and-fire (LIF) neurons was proposed (Zamarreño-Ramos et al., 2011; Serrano-Gotarredona et al., 2013). However, a conceptual demonstration of realistic memristor synapses for communication and learning has not been achieved so far.

This work addresses the integration of memristors in neuromorphic systems by introducing a 2-transistor/1-resistor (2T1R) synapse for large scale neuromorphic systems. The transistors in the synapse block allow for (i) multiple-input control of the synapse, which must receive signals from both the PRE and the POST, and (ii) accurate control of the filament growth for analog switching and STDP behavior (Yu et al., 2011; Ambrogio et al., 2013; Subramaniam et al., 2013). STDP in the 2T1R synapse is experimentally demonstrated on bipolar resistive switching memories based on HfO_2_ acting as memristive switches. We show that the memristive synapse is capable of communication of spiking signals between neurons and stochastic STDP due to both the natural switching variability in the switch, and to the variations of memristive response depending on the initial state. We finally show a conceptual demonstration of a simulated 2-layer neuromorphic network displaying stochastic pattern learning and recognition, thus further supporting memristive synapse as a scalable, high-functionality building blocks for large scale neuromorphic systems.

## Materials and methods

**Figure 1:**
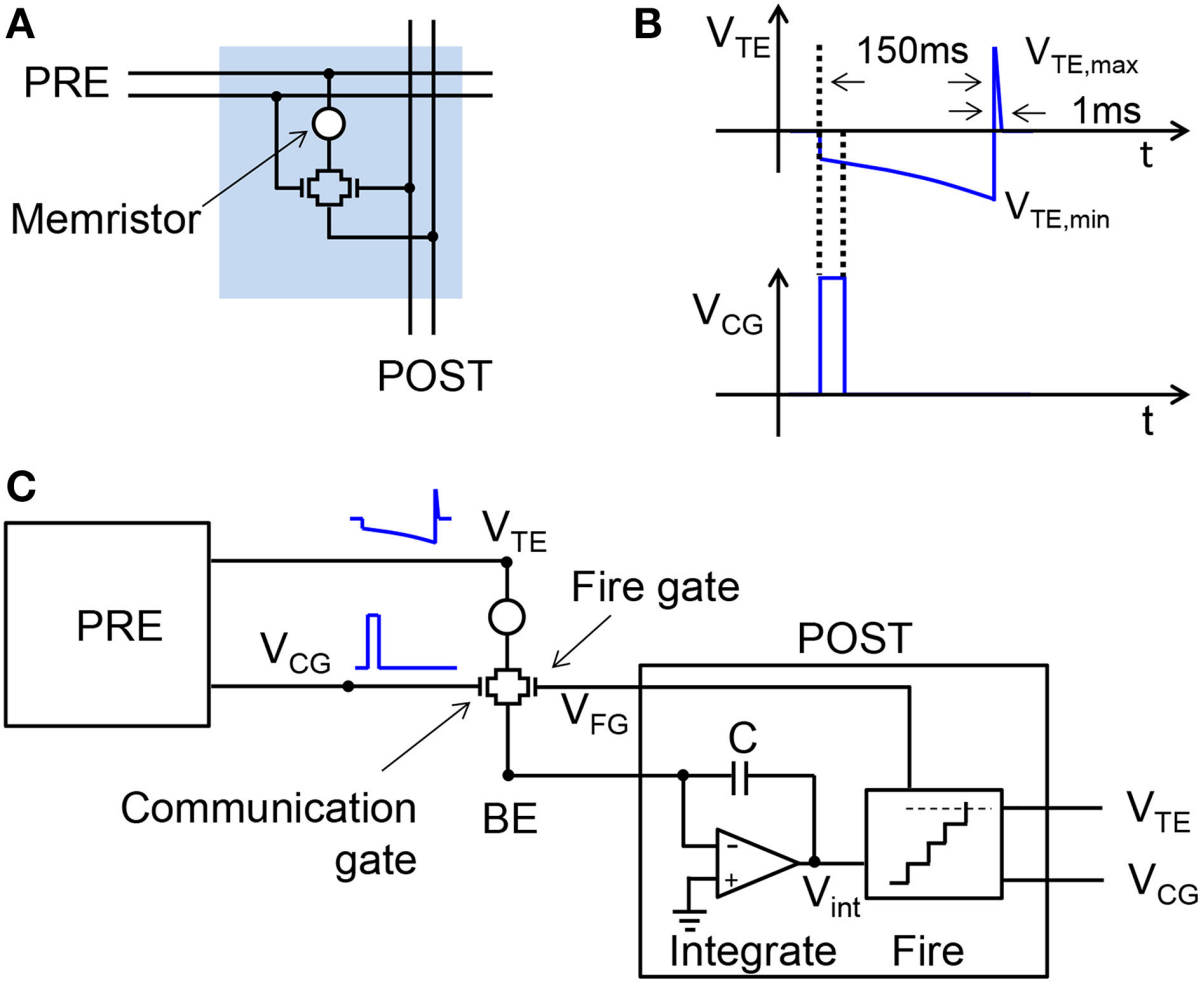
**Illustrative scheme for the 2T1R synapse and its operation**. The synapse consists of a memristor with 2 series transistors, connected to both the PRE and POST **(A)**. During communication, the PRE delivers pulses to both the CG and the TE terminals of the synapse **(B)**. The resulting current is a function of the memristor conductance and is fed into the input node of the integrate-and-fire POST neuron **(C)**. The maximum and minimum voltages of TE pulse are V_TE,max_ = 2.4 V and V_TE,min_ = −1.65 V, respectively.

Figure 1A shows the conceptual scheme of the 2T1R structure for the memristive synapse. Both MOS transistors in the synapse control the current flowing through the memristor, thus enabling communication and plasticity. The PRE controls 2 of the 4 terminals of the 2T1R structure, namely the top electrode (TE) and the communication gate (CG). The bottom electrode (BE) is instead connected to the virtual-ground input of the POST, which also controls the fire-gate (FG) terminal.

### Communication mode

The usual operation of the synapse consists of the communication mode, where the synapse is a simple resistor with fixed conductance allowing for the weighted transmission of spikes from the PRE to the POST (Zamarreño-Ramos et al., 2011; Indiveri et al., 2013). Figure 1B shows the waveforms of the pulses applied to the TE and the CG. The TE pulse includes an exponentially-increasing negative pulse and a short positive pulse, while the CG pulse is a short positive pulse enabling the transmission of a negative current pulse to the POST input through the BE connection. Although the CG voltage is high, it always overlaps with the low-voltage region of the V_TE_ pulse, which rules out any possible switching in the memristor. The negative current spike is integrated by the input stage of the POST as shown in Figure 1C, illustrating a single PRE/synapse/POST layer of the neuromorphic network. The integrate-and-fire structure of the POST in Figure 1C is largely simplified, in that it does not include, e.g., the leakage path for the stored charge, the refractory period to deactivate integration during fire, and the reset switch to initialize integration after fire (Zamarreño-Ramos et al., 2011). As the PRE spikes collected at the neuron input are integrated, the internal voltage V_int_ increases, eventually hitting the threshold of the comparator stage. This event triggers the fire circuit, namely a monostable circuit delivering spikes in the forward direction, i.e., to the TE and CG terminals of the output synapse, and in the reverse direction, i.e., to the FG terminal of the input synapses.

### STDP

**Figure 2:**
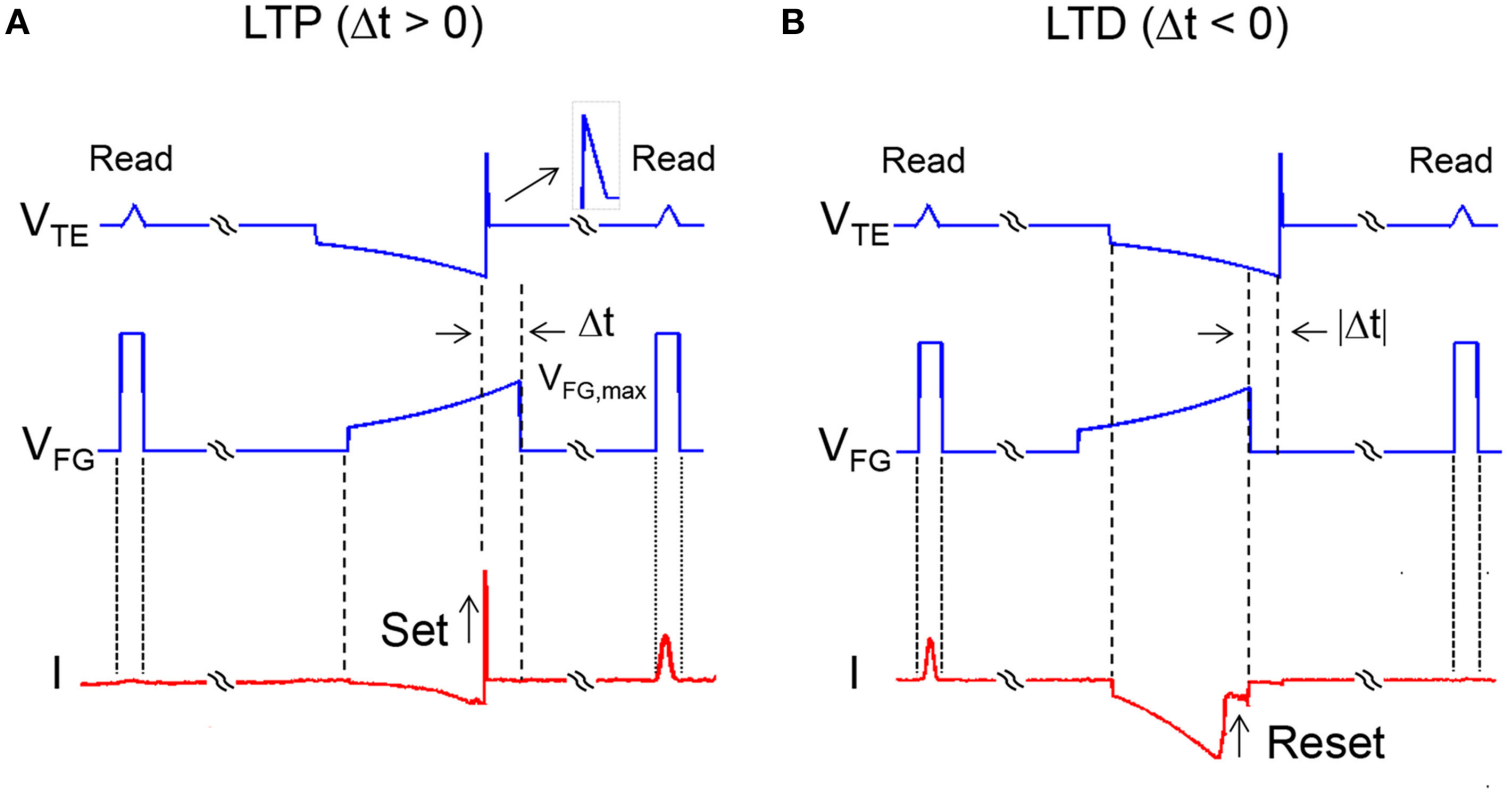
**Signal waveforms during LTP and LTD**. LTP takes place when the delay Δ*t* between V_TE_ and V_FG_ is positive **(A)**. In this case, there is overlap between the positive 1-ms TE pulse and the FG pulse (maximum voltage 2.9 V), thus inducing set controlled by the V_FG_-value. V_FG_ increases at decreasing Δ*t*, thus the maximum LTP is obtained for Δ*t* approaching 0. LTD takes place when the delay Δ*t* between V_TE_ and V_FG_ is negative **(B)**. In this case, the negative TE pulse and the positive FG pulse overlap each other, thus inducing reset controlled by the V_TE_-value. V_TE_ increases in absolute value at decreasing Δ*t*, thus the maximum LTD is obtained for Δ*t* approaching 0.

The temporal coincidence of the PRE spike at the TE of a synapse and of the POST spike (or fire) at the FG of a synapse leads to a change of the memristor conductance according to Figure 2. Two cases can be distinguished by the relative delay Δ*t* defined as the time between the end of the negative TE pulse and the end of the FG pulse. For Δ*t* > 0 in Figure 2A, there is an overlap between the positive 1-ms TE pulse and the FG pulse, thus inducing set transition in the memristor. The increase of conductance, due to the growth of a conductive filament (CF) across the HfO_2_ switching layer (Nardi et al., 2012), is dictated by the compliance current I_C_ flowing in the transistor, hence by the gate voltage V_FG_. Since the FG voltage V_FG_ decreases at increasing Δ*t*, LTP decreases as Δ*t* increases, thus realizing a timing-dependent LTP. Figure 2B also includes triangular read pulses at V_TE_ before and after the PRE and POST spikes, both having 1 ms width and a small amplitude of 0.5 V to avoid any disturb to the memristor device. A rectangular pulse of width 1 ms and amplitude 5 V was applied to V_FG_ to enable the pulse operation. The response current during the read pulse before and after the PRE/POST spikes allows to evaluate the increase of conductance induced by LTP.

Similarly, for Δ*t*<0 (Figure 2B), the negative TE pulse and the positive FG pulse overlap each other, thus inducing reset transition due to the disconnection of the CF. The increase of resistance during reset is controlled by the maximum voltage across the memristor (Nardi et al., 2012), hence by the value of V_TE_. Since V_TE_ decreases in absolute value at increasing Δ*t*, LTD decreases with Δ*t*, thus carrying out time-dependent LTD. The combination of time-dependent LTP and LTD results in STDP functionality.

### Circuit implementation

To verify the conceptual scheme of STDP in Figure 2, we applied the waveform in the figure to a 1T1R structure including an HfO_2_ memristor in series with a MOS transistor. The MOS transistor has threshold voltage *V*_T_ = 1.4 V, while the channel width and length were 3 μm and 1.425 μm. In the memristor, a Si-doped HfO_2_ layer was sandwiched between two TiN electrodes. A Ti cap was deposited between the top TiN electrode and the HfO_2_ layer to allow for oxygen extraction aimed at the formation of a local sub-stoichiometric HfO_x_ (*x* < 2) layer close to the top electrode. This oxygen-exchange layer (OEL) is believed to act as a defect reservoir for the injection during the set transition, when the positive applied voltage induces migration of defects, such as oxygen vacancies and metallic impurities (Hf, Ti) responsible for the formation of a conductive channel, thus resulting in a relatively low resistance. The application of a negative voltage instead results in the retraction of the conductive channel back toward the OEL, thus leading to a relatively high resistance. The HfO_2_ layer had an amorphous structure after deposition. The HfO_2_ thickness was 10 nm, while the Ti cap thickness was 15 nm. More details about the experimental samples are reported elsewhere (Ambrogio et al., 2014a; Calderoni et al., 2014). The CG transistor was not connected in the experiment, due to our focus on demonstrating STDP. Figure 1C shows the conceptual scheme of the 2T1R structure for the memristive synapse. The FG pulse had extreme voltages of 2.9 and 1.0 V, with time constant τ = 140 ms. The same time constant was used for the exponential region of the TE pulse, where the extreme voltages were −1.65 and −0.55 V. The 1-ms half-triangle positive pulse had an extreme amplitude of 2.4 V.

## Results

### Experimental STDP characteristics

**Figure 3:**
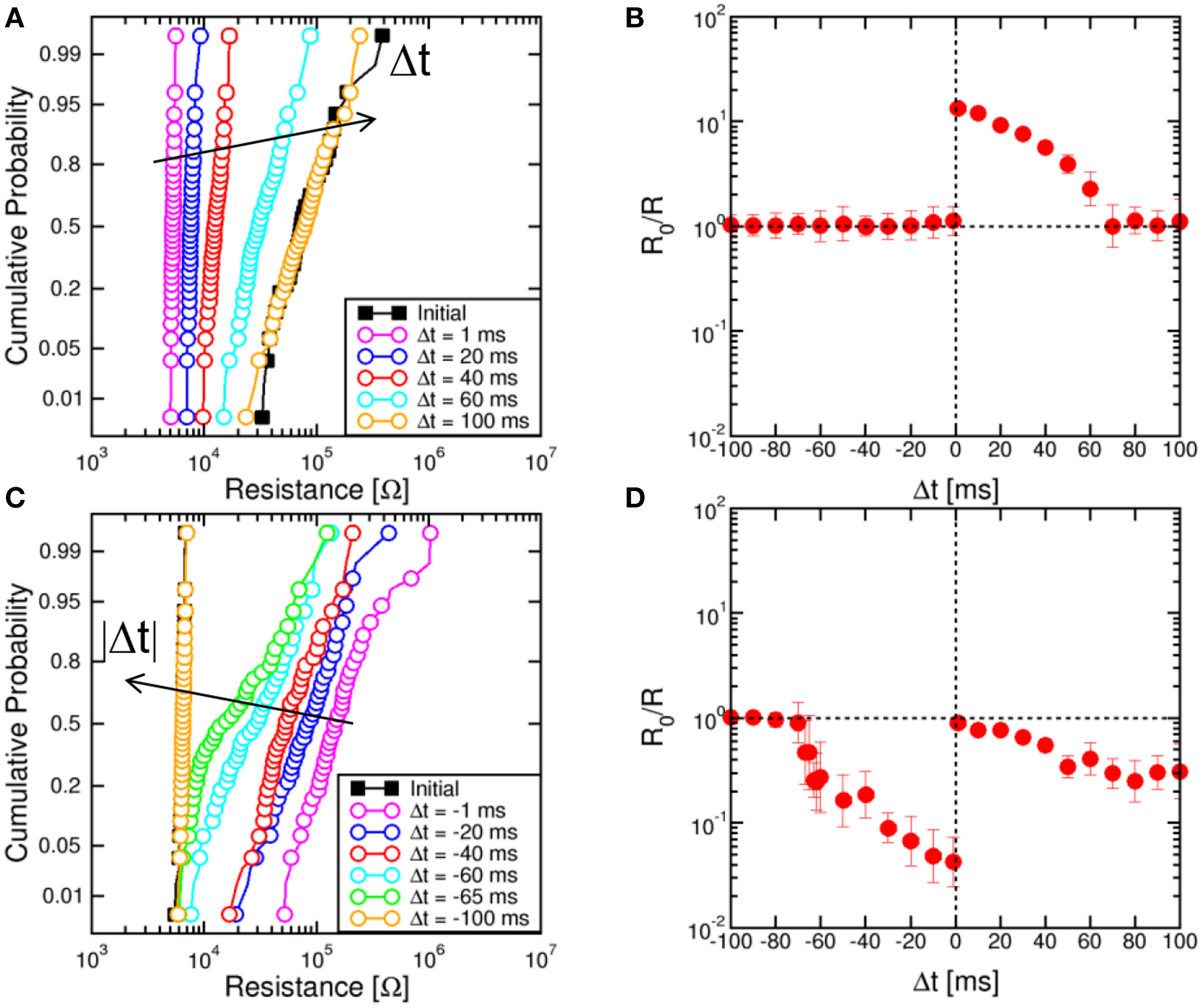
**Cumulative distributions of R for variable Δ*t* and corresponding STDP characteristics**. Cumulative distributions for Δ*t* > 0 show an increasing R for increasing Δ*t*, starting from a high-resistance state (R_0_ = 100 kΩ) of the memristor **(A)**. Correspondingly, the conductance change R_0_/R decreases at increasing Δ*t* in the STDP characteristic **(B)**. Similarly, for LTD starting from a low-resistance state (R_0_ = 5 kΩ) of the memristor, the cumulative distributions show that R decreases at increasing negative delay **(C)**, while the conductance change R_0_/R decreases for large delays in the STDP characteristic **(D)**.

Figure 3A shows the cumulative distributions of measured resistance R in the memristor after application of TE and FG pulses at increasing Δ*t*. The same STDP experiment with a given Δ*t* was repeated 100 times to allow for a sufficient statistical accuracy. The device was always prepared in a full reset state, corresponding to a resistance of about 100 kΩ, and the delay Δ*t* was changed between 1 and 100 ms. The distributions show a decreasing value of R at decreasing delay, in agreement with the expected time-dependent LTP in Figure 2A. Figure 3B summarizes the conductance enhancement R_0_/R, where R_0_ is the initial resistance and R is the median value of the distribution. The figure shows time-dependent increase of conductance (LTP) for Δ*t* > 0, while no change of resistance is obtained for Δ*t*<0. Figure 3C shows the cumulative distribution of measured R for negative Δ*t* in the range between −1 and −100 ms. To demonstrate LTD, the memristor was initialized in a low resistance state with R_0_ around 5 kΩ, obtained with a pulse of 1 ms at *I*_C_ = 170 μA. Figure 3D shows the conductance change R_0_/R indicating time-dependent LTD for Δ*t*<0. LTD can also be seen at positive delays, which is due to a sequence of reset and set events in the memristor during the negative and positive regions of the TE pulse, respectively. First, a reset transition takes place due to the negative V_TE_, then the 1-ms V_TE_ pulse induces a set transition with relatively low I_C_. As a result, the device is in a set state finally, although with smaller conductance than the initial state, due to the relatively small I_C_. Since I_C_ decreases at increasing positive Δ*t*<0, LTD increases with Δ*t*.

Distributions in Figures 3A,C show a significant variability, although they were obtained by repeating the same experiments several times on a single device. The distribution variance can be attributed to the switching variability in memristive devices, which was shown to result from the discrete number of defects in the CF (Ambrogio et al., 2014a). The natural switching variability ensures stochastic plasticity in the artificial synapse, where the final state is not deterministically dictated by Δ*t*, rather it is affected by an inherent standard deviation. Note that the relative spread increases with R in Figure 3, due to the decreasing number of defects and the correspondingly large statistical spread (Ambrogio et al., 2014a).

**Figure 4:**
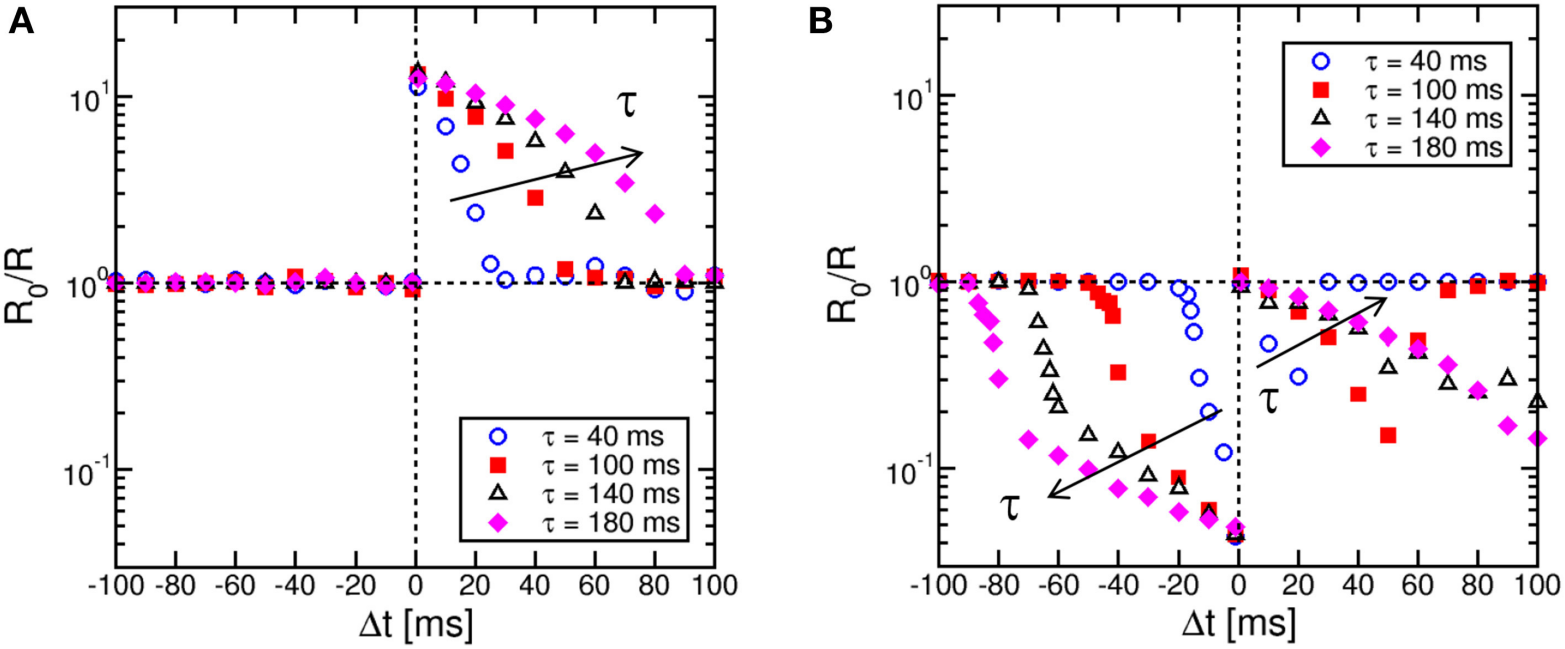
**STDP characteristics at increasing time constant τ**. The STDP characteristics stretch to longer Δ*t* as the time constant describing the V_TE_ pulse increases, for both LTP on high-resistance states **(A)** and LTD on low-resistance states **(B)**.

Figure 4 shows STDP characteristics for variable time constant τ in the range between 40 ms and 180 ms, for the memristor initially prepared in a high resistance state (a) or a low resistance state (b). LTP (a) and LTD (b) characteristics show the same behavior as in Figure 3, except for a stretching along the Δ*t* axis for increasing τ as a result of the change of the slope of the exponential TE and FG pulses. These results demonstrate the tunability of the STDP characteristics on the timescale through a proper choice of the time constant.

#### Dependence on the initial state

**Figure 5:**
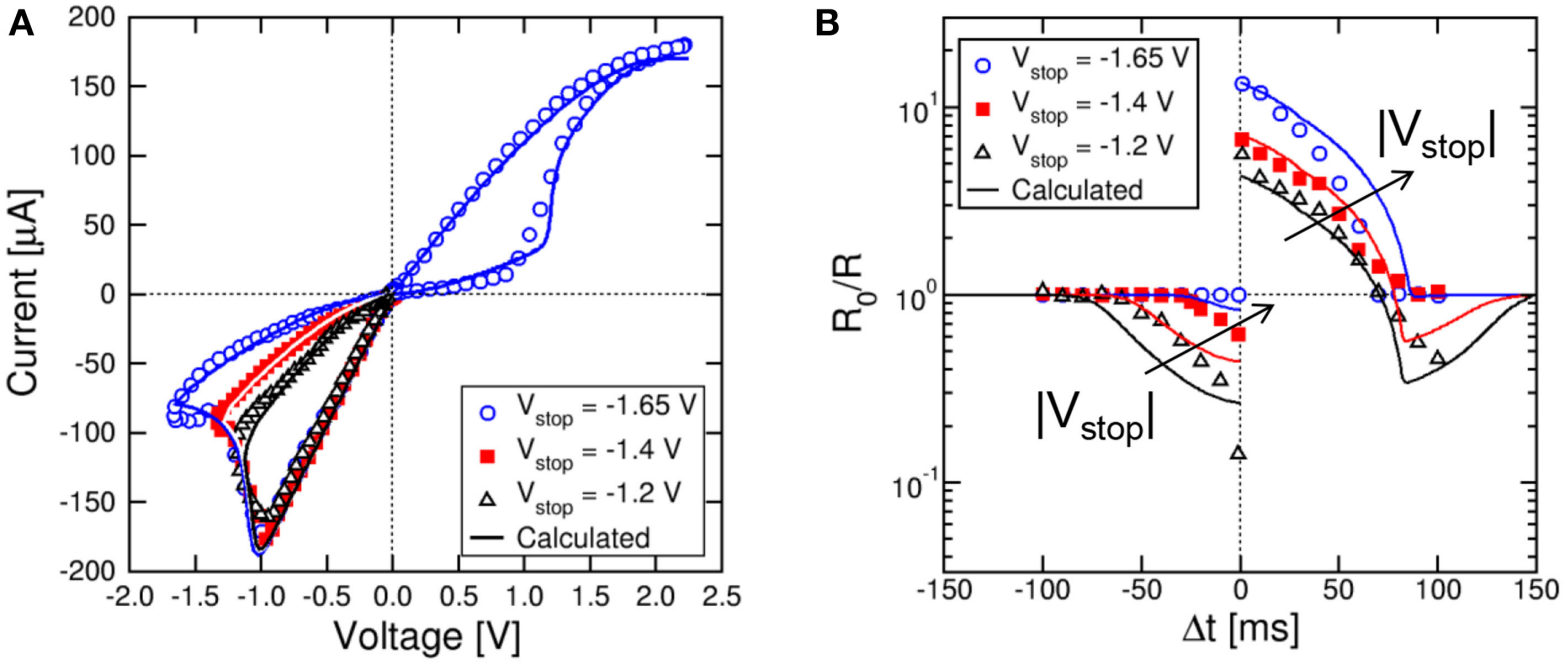
**STDP response at variable high-resistance states**. Variable high-resistance states are obtained by resetting the memristor device at increasing negative voltage V_stop_ as shown in the I–V curve **(A)**. The STDP characteristics show increasing LTP and decreasing LTD at increasing initial R **(B)**. Analytical calculations well account for the experimental data as a function of V_stop_.

While results in Figures 3, 4 were obtained for the memristor initialized in either the high resistance (for LTP) or the low resistance state (for LTD), it is important to demonstrate the functionality of the STDP scheme for any arbitrary initial state. We first considered variable reset states, obtained by first setting the device to a reference initial low resistance state with a compliance current *I*_C_ = 170 μA, then resetting the device with a variable maximum negative voltage V_stop_, as shown in Figure 5A. Here, the set and reset transitions in the HfO_2_ memristor can be seen at positive and negative voltage, respectively. As the reset voltage increases, the resistance increases, as a result of the increasing growth of the depleted gap along the CF (Nardi et al., 2012). The memristor resistance values were 25, 45, and 100 kΩ for V_stop_ equal to −1.2, −1.4, and −1.65 V, respectively. Also shown are simulation results according to our physics-based analytical model for resistive switching devices (Ambrogio et al., 2014b). In this model, the Fourier equation for heat generation and conduction is analytically solved, then the local temperature at the injecting point along the CF is used to estimate the migration rate and the corresponding change of CF diameter (during set transition) and depleted gap (during reset transition). The energy barrier controlling ion migration in the analytical model was *E*_A_ = 1.2 eV. Simulation results in Figure 5A support the model as an accurate tool for predicting real memristive switching in metal oxide systems.

Figure 5B shows the measured and calculated STDP characteristics for variable high resistance states in Figure 5A. As the initial resistance R_0_ increases, the LTP conductance change increases, while the LTD conductance change decreases. However, the shapes of LTP and LTD characteristics are qualitatively the same irrespective of the R_0_.

**Figure 6:**
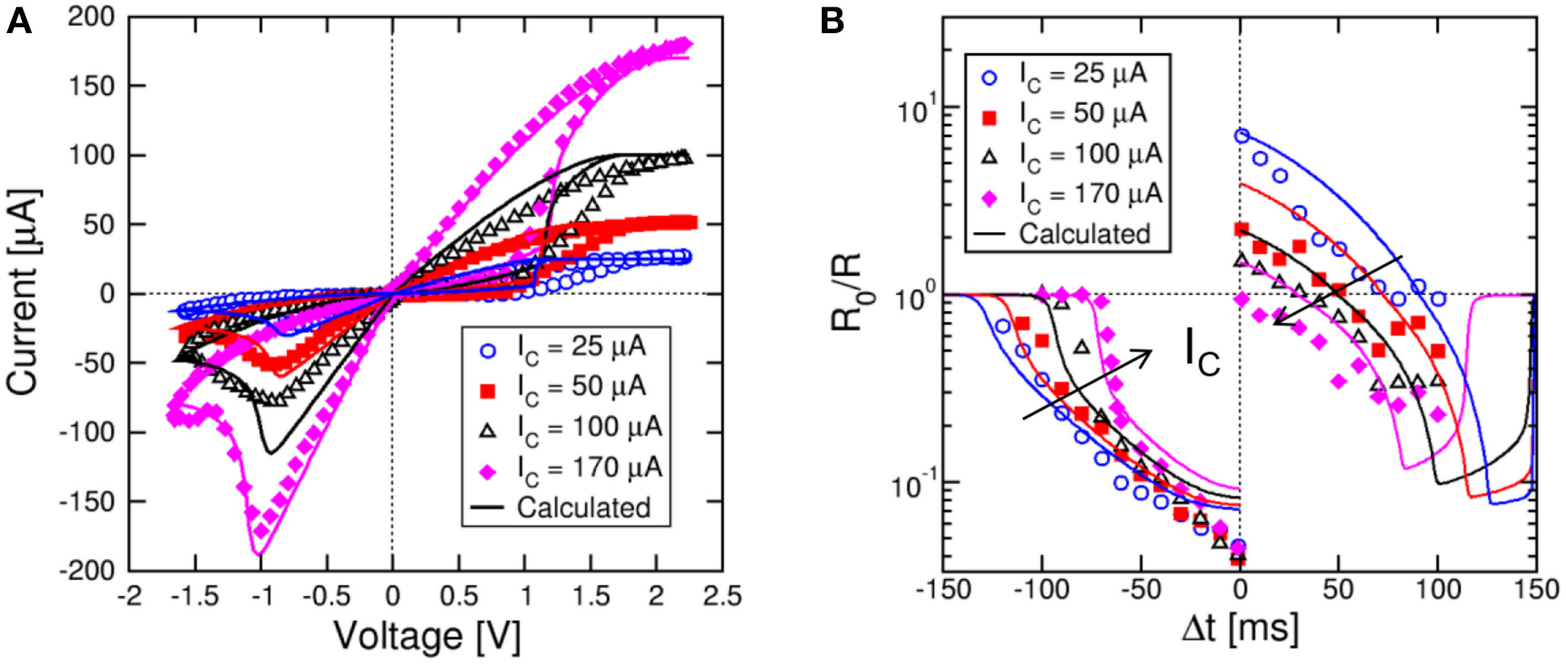
**STDP response at variable low-resistance states**. Variable low-resistance states are obtained by setting the memristor device at increasing compliance current I_C_ as shown in the I–V curve **(A)**. The STDP characteristics show increasing LTP at increasing initial R, while LTD characteristics change only slightly **(B)**. Analytical calculations well account for the experimental data as a function of I_C_.

Similarly, we studied variable set state, namely state obtained with variable compliance current during set. Figure 6A shows the measured and calculated I–V curves for *I*_C_ = 25, 50, 100, and 170 μA. Both set transition at positive voltage and reset transition at negative voltage are shown in the figure. Simulations by the analytical model again accounts closely for the experimental behavior. As I_C_ increases, the set state resistance decreases, as a result of the larger diameter of the CF (Nardi et al., 2012). Note that the reset current I_reset_ is approximately equal to I_C_ (Kinoshita et al., 2008; Lee et al., 2008), while the reset voltage V_reset_ is approximately constant around 1 V, marking the voltage needed to initiate defect ionization and migration within the CF (Ielmini, 2011). Figure 6B shows the measured and calculated STDP characteristics for variable initial low-resistance state as in Figure 6A. Calculations again provide a satisfactory agreement with data and can predict the state-dependent learning in the synapse.

The STDP characteristics in Figures 5, 6 show LTD at both positive and negative Δ*t*, which disagrees with the standard timing-dependence of biological learning (Bi and Poo, 1998). However, it was shown that biological synapses might have diversified response based on their function and typologies (Abbott and Nelson, 2000). For instance, a similar STDP response with LTD at positive Δ*t* was observed in hippocampal CA1 neurons (Nishiyama et al., 2000; Wittenberg and Wang, 2006) and explained as due to the Ca^+^ dynamics (Caporale and Dan, 2008). This demonstrates that the memristive STDP response in 2T1R synapse is compatible with learning functions in biological neural networks.

#### Stochastic learning

Results in Figures 5, 6 suggests that, for any given Δ*t*, the potentiation/depression of the synapse also depends on the initial state, which introduces a certain degree of stochastic response in the STDP characteristics. To study the stochastic behavior of STDP, we performed experiments with a sequence of coupled TE and FG pulses as in Figure 2, applied to the same synapse initially prepared in a high resistance state. A total number of 55 different sequences were applied, each including 10 spikes with randomly chosen Δ*t*. Each random sequence was repeated 50 times to achieve sufficient statistical significance. The time constant was 140 ms in all experiments and simulations.

**Figure 7:**
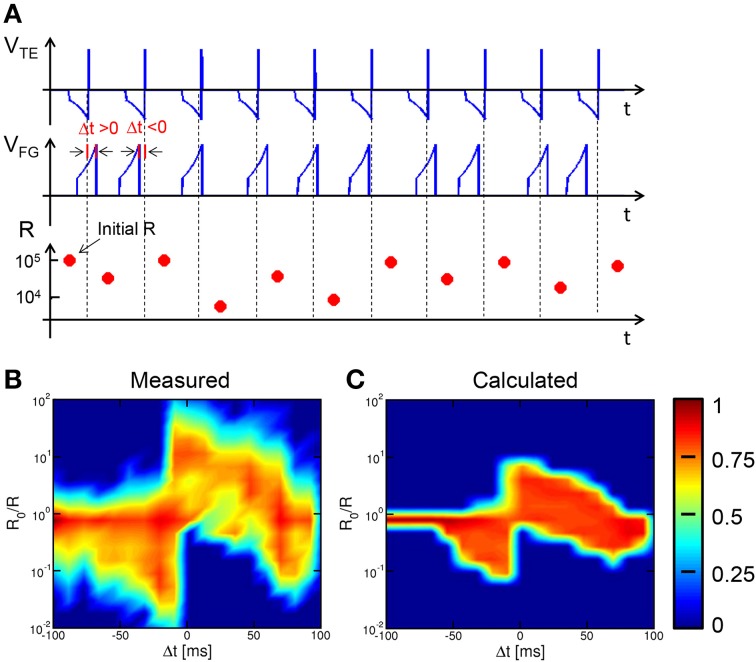
**STDP over a random sequence of spikes**. A sequence of partially-overlapping PRE/POST spikes with random Δ*t* are applied to the synapse, resulting in LTP or LTD depending on the relative delay **(A)**. The conductance change R_0_/R has been collected over 50 repeated experiments with 55 different sequences, each containing 10 random spikes. For any Δ*t* and R_0_/R, the probability has been reported in colour scale **(B)**. Calculated results show similar stochastic STDP characteristic **(C)**.

Figure 7 shows (from top to bottom) the V_TE_ waveform, the V_FG_ waveform and the corresponding resistance R for a random sequence of 10 spikes. Read pulses similar to the waveform in Figure 2 (not shown in Figure 7A) were applied after each spike to measured R. Figure 7B shows the color map of the occurrence of any value of conductance change R_0_/R as a function of Δ*t* for all 27,500 random spikes. The ratio R_0_/R was defined as the ratio between resistances before and after the STDP event. The maximum probability (red) indicates LTD for negative Δ*t* and for relatively large positive Δ*t*, while LTP occurs for relatively small positive Δ*t*. Figure 7C shows the color map of R_0_/R as a function of Δ*t* for 10^4^ simulated sequences assuming random Δ*t* and using the same analytical switching model for the memristor as in Figures 5, 6. The calculated color map shows a qualitative agreement with the experimental STDP, indicating potentiation at small Δ*t* > 0, and depression at negative Δ*t* and large positive Δ*t*. The STDP statistics, where different LTP and/or LTD are obtained for any given Δ*t*, is mainly due to the dependence on the initial state as discussed in Section Experimental STDP Characteristics Experimental data in Figure 7B indicate a larger spread of R_0_/R, which we attribute to the additional source variability due to the naturally stochastic switching, i.e., the physical origin of the distribution spread in Figures 3A,C.

**Figure 8:**
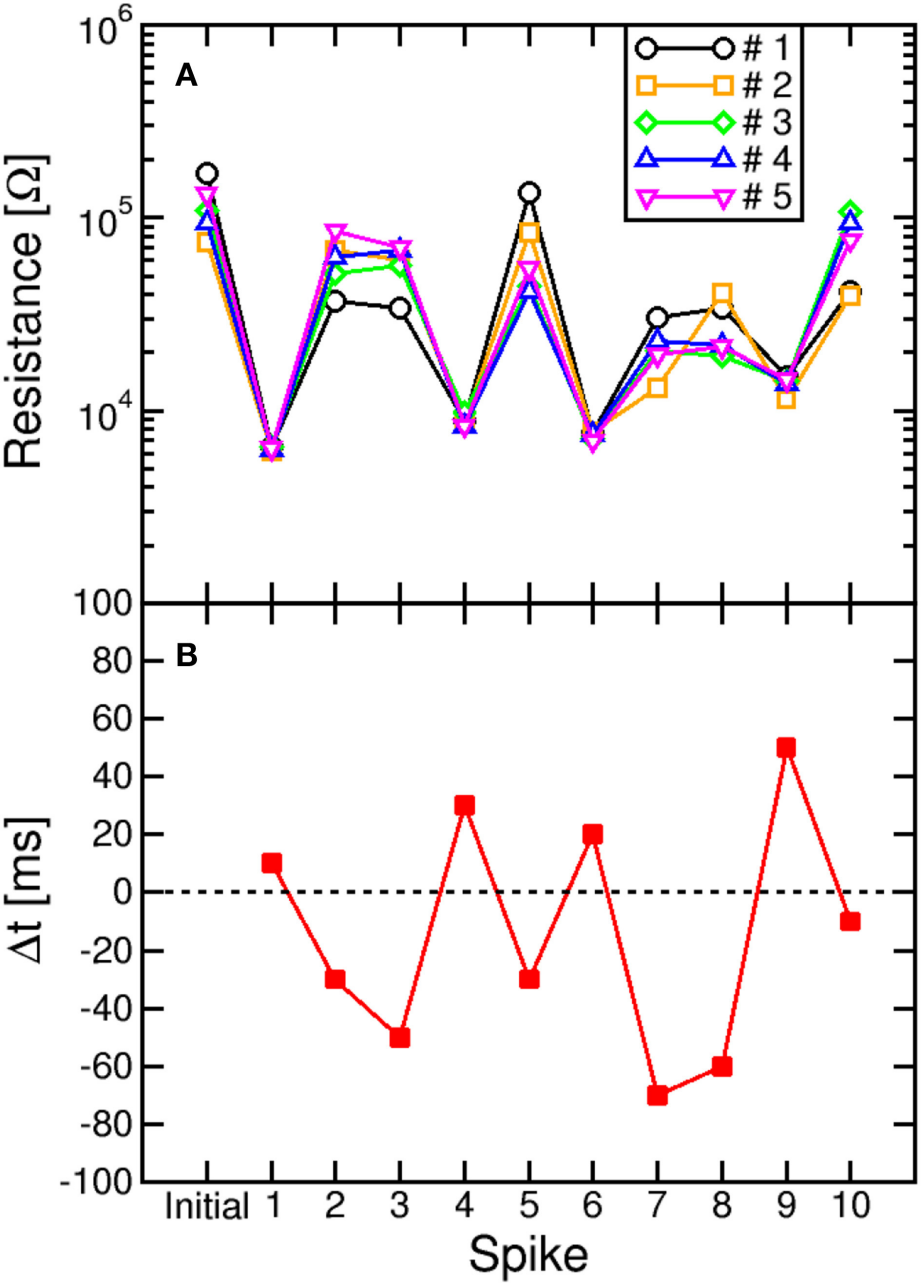
**Stochastic LTP and LTD**. The resistance **(A)** was plotted as a function of the number of the spike within a sequence with determined delay Δ*t* **(B)**. The sequence was repeated 5 times to highlight the variability of resistance change during LTP and LTD. natural switching variability leads to random walk of R during each set/reset operation, with reset (LTD) process showing generally larger stochastic variation compared to set (LTP) process.

The impact of switching variability is also highlighted in Figure 8, showing the values of R measured after each spike in a sequence of 10 events with random timing Δ*t*. Figure 8A compares 5 typical sequences always starting from the same initial high resistance state (about 10^5^ Ω), to study the effect of switching variability. The measured R displays random walk depending on Δ*t*, which is shown in Figure 8B. Note the significant random change among all trajectories due to the stochastic switching during each set/reset operations. The largest variability is seen for LTD, due to the large variability in the high resistance state (see, e.g., Figures 3A,C). On the other hand, LTP leads to a certain decrease of variability, since the set operation is mainly controlled by I_C_ and negligibly depends on the initial high-resistance state (Ambrogio et al., 2014a).

### Patterning learning and recognition through STDP

**Figure 9:**
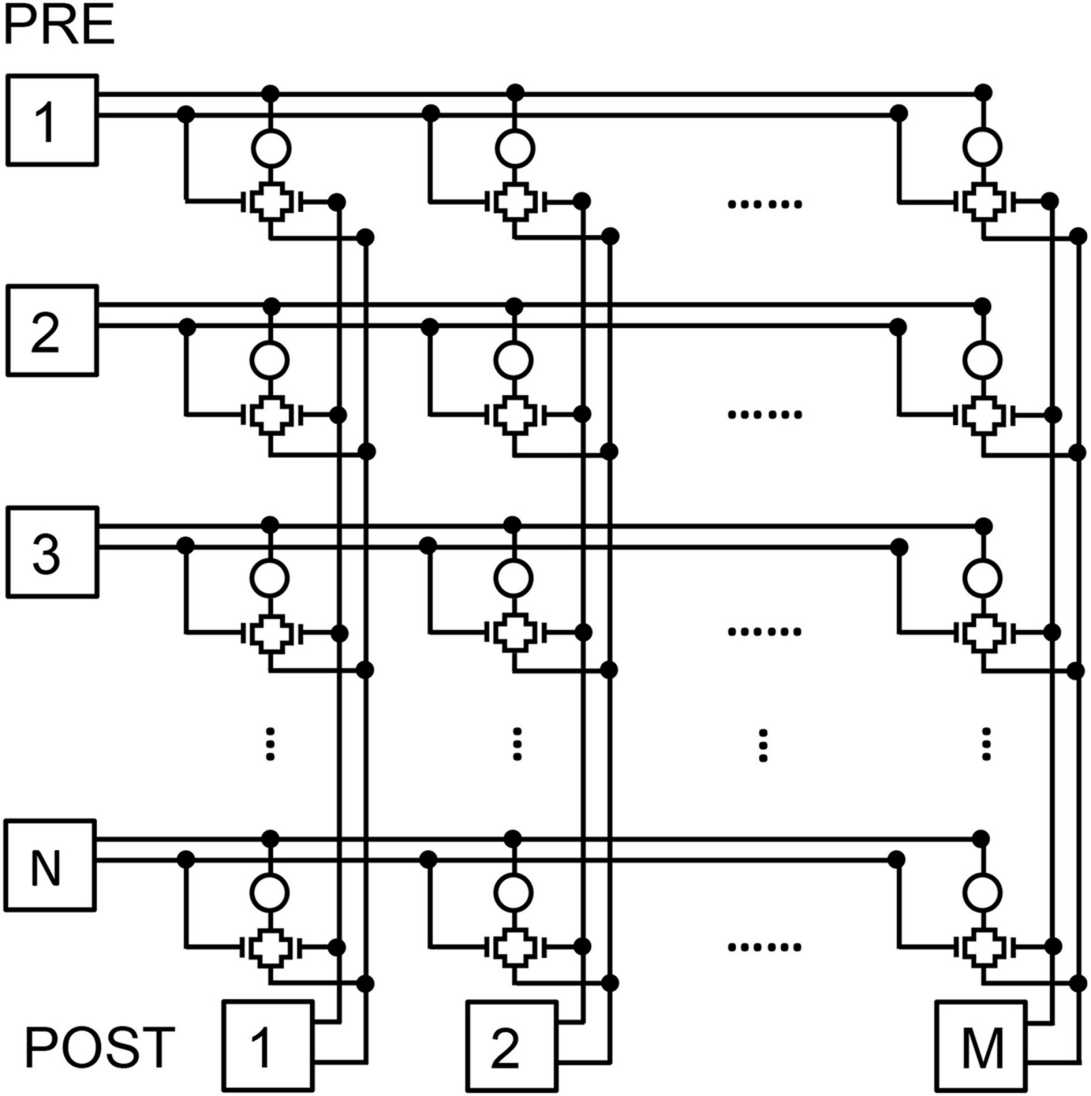
**Schematic illustration of the 2-layer neuromorphic network**. The first layer consists of N PRE, while the second layer consists of M POST, thus resulting in a network of NxM synapses with 2T1R structure.

To verify that STDP in the 2T1R synapse is capable of pattern learning and recognition, we adopted a 2 layer neuromorphic network schematically shown in Figure 9. Here, N pre-synaptic neurons provide spiking input to M post-synaptic neurons through an array of NxM synapses (Zamarreño-Ramos et al., 2011). Connections to PRE and POST in Figure 9 are organized according to rows and columns, respectively, each requiring 2 lines for connecting the 2T1R synapse, namely the TE and CG line from PRE to the synapse and the BE and the FG between the synapse and the POST.

**Figure 10:**
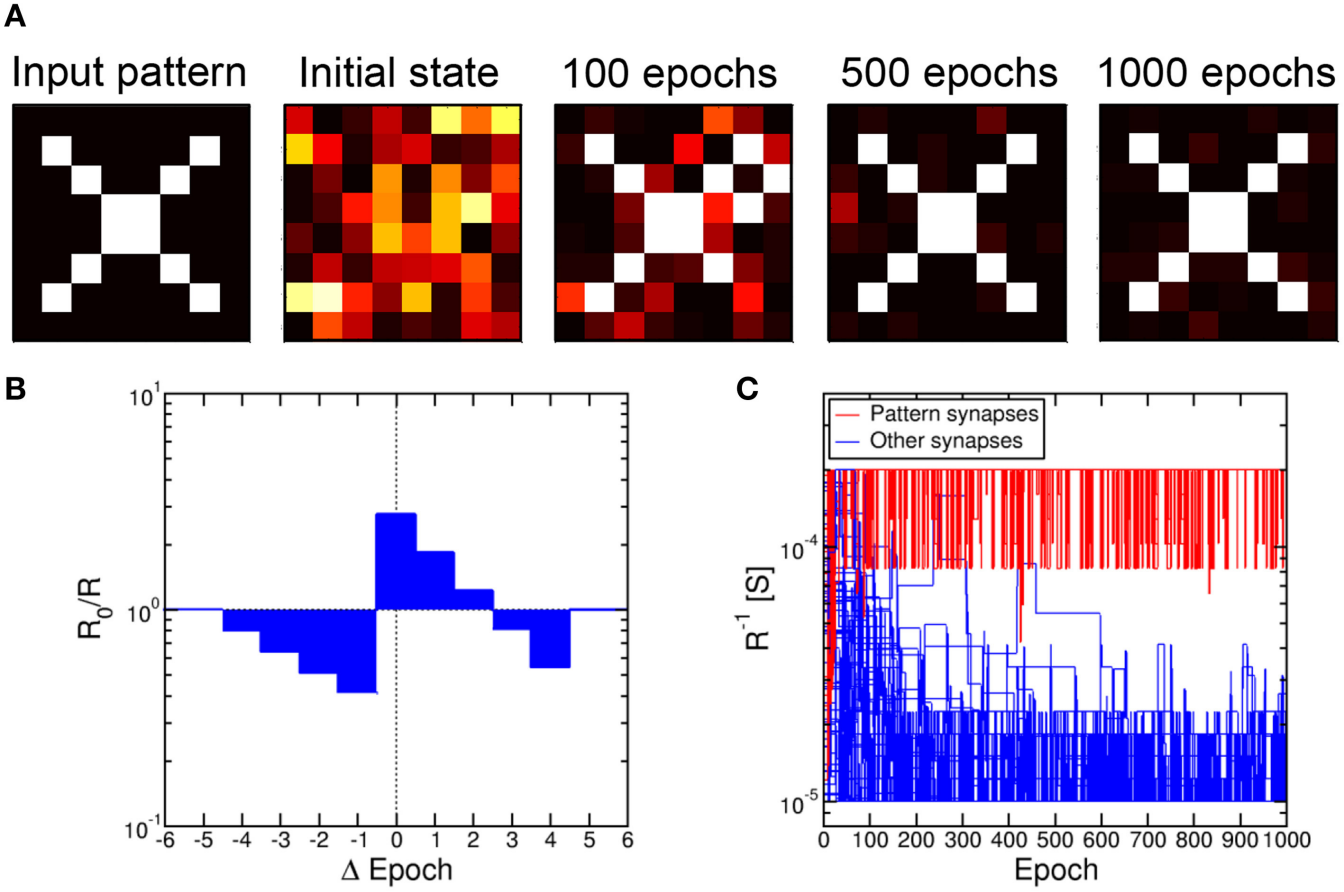
**Pattern learning and recognition through 2T1R synapses**. The input pattern was fed by the first layer of 8 PRE neurons toward a second layer of 8 POST neurons, resulting in learning as demonstrated by the evolution of the synapse weights **(A)**. Each synapse was changed according to a simplified STDP characteristic with discrete delay **(B)**. The conductance of pattern synapses increases due to the learning process, while other synapses experience increasing depression **(C)**.

To simulate pattern learning, we assumed that the N PRE neurons belong to an artificial retina providing visual stimuli corresponding to the 8 × 8 square pattern at the extreme left in Figure 10A (*N* = 64). The pattern was fed synchronously from PRE to POST through the synapse array, by applying a spike for every white pixel while black pixel did not yield any spike. The pattern was randomly alternated with random noise, consisting of 95% probability for black and 5% for white signals in each of the N pixels. The duty cycle of true pattern occurrence was 50%. All signals received at a POST were integrated according to the scheme in Figure 1C, then a fire signal was triggered as the internal potential V_int_ reached a given threshold. The fire signals were delivered from the POST to all connected synapses, and dictated a conductance change according to the simplified STDP characteristic in Figure 10B. This includes LTP for small Δ*t* > 0 and LTD for Δ*t* < 0 and for large Δ*t* > 0, according to the most general response of the 2T1R synapse in Figures 5, 6. As a minimum resistance *R* = 5 kΩ was reached, further potentiation was inhibited in the synapse, while depression was inhibited above a resistance *R* = 100 kΩ.

Figure 10C shows the calculated conductance 1/R for 64 synapses in a single column, which connected all PRE to a single POST. Starting from a uniformly distributed random initial state, the synapse conductance, or weight, generally follows 2 trends, either increasing or decreasing with time due to repeated LTP and LTD. The evolution of the synapse weights is also shown in Figure 10A for 4 states, namely initial state and after 100, 500, and 1000 epochs of pattern presentation. The pattern is seen to rapidly potentiate the corresponding synapses, with potentiation and depression occurring in white and black pixel positions, respectively. On the other hand, a longer time is needed for depression of unstimulated synapses, since depression relies on uncorrelated random noise patterns. While potentiation of pattern synapses takes about 30 epochs, the depression of other synapses is completed in about 500 epochs. These results fully support the capability for pattern learning and recognition by the scheme in Figure 2, combined with the STDP response of our 2T1R synapse which was simplified in Figure 10B.

**Figure 11:**
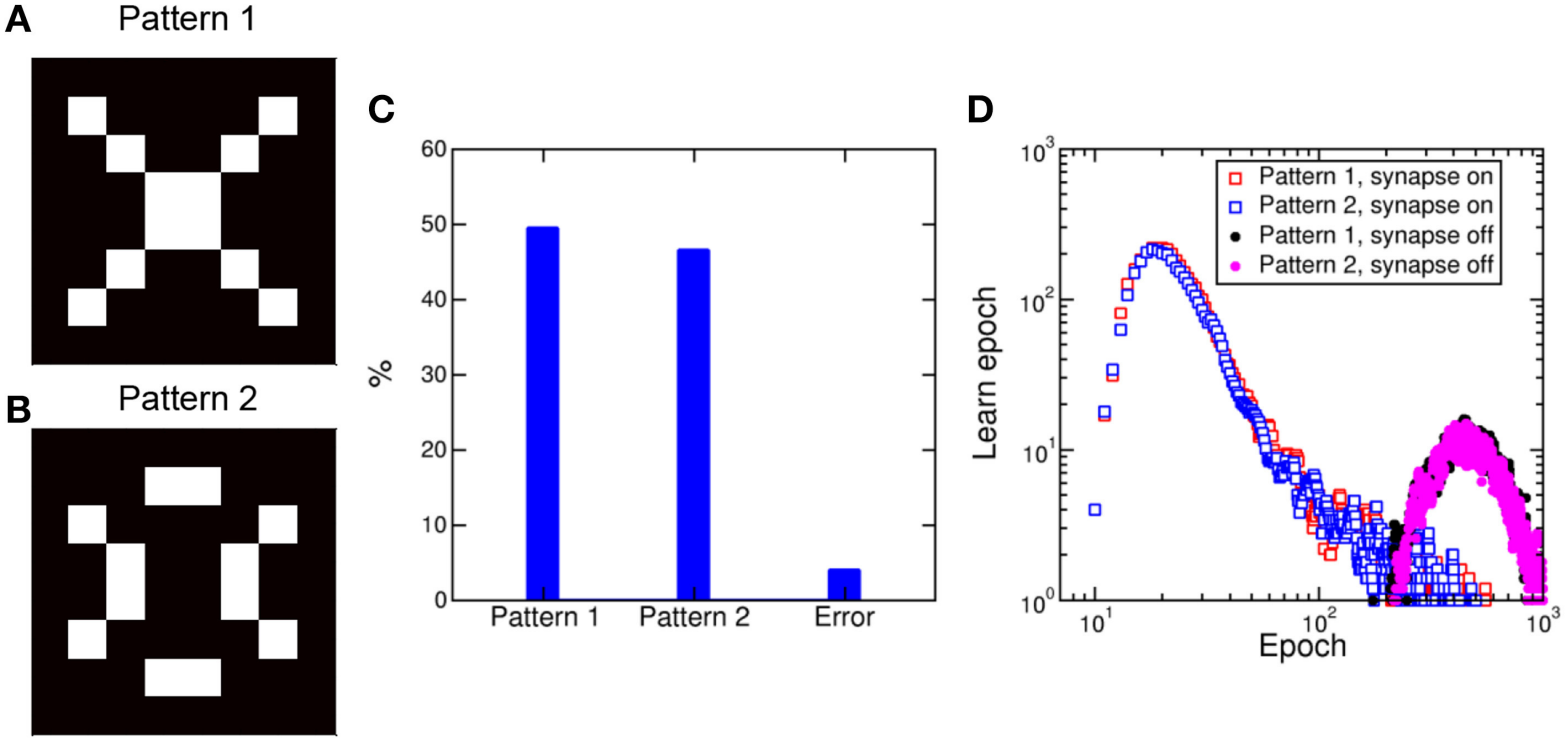
**Pattern competition during learning**. Random submission of pattern 1 **(A)** and pattern 2 **(B)** in a 8 × 8 synapse array results in learning of either pattern with equal probability approaching 50%, including a minority of error due to transition from one pattern to the other **(C)**. Potentiation of pattern synapses takes place in about 20 epochs, while depression of out-of-pattern synapses requires around 500 epochs **(D)**.

A 2-layer network similar to Figure 9 was previously shown to lead to random specialization of POST neurons to distinct patterns, such as the cars appearing in specific lanes on the highway (Bichler et al., 2012). We verified the random specialization in our system by considering a NxM network as in Figure 9 with *N* = 64 (number of pixels in the pattern and number of PRE neurons) and *M* = 10,000 (number of POST neurons). We presented the 2 patterns in Figure 11A and b in a random sequence of patterns (70% probability equally distributed between pattern 1 and 2) and random noise (30% probability). The initial values of the synapses were randomly distributed as in Figure 10. Figure 11C shows the percentage distributions of patterns recognized after a total number of 10^3^ epochs: Patterns 1 and 2 were recognized with equal probability of about 48%, while no recognition was possible in 4% of the cases. Most of these recognition failure are due to incorrect recognition of the two patterns, converging to a mixture of patterns 1 and 2, while some errors are due to very slow learning, leading to incomplete learning at the final calculated epoch. Figure 11D shows the probability distributions for potentiating, hence learning, pattern 1 and 2, identified as the first epoch with all synapses completely potentiated. Both distributions peak at about 20 epochs, with no preference for any of the 2 patterns. Note that the patterns 1 and 2 were selected to have the same number of black/white pixels, to ensure a constant average firing rate of the POST. This accounts for the equal learning times in Figure 11C. Figure 11D also shows the distribution of times corresponding to the depression of all the synapses not belonging to pattern 1 or 2. The distributions show a similar behavior and peak at 500 epochs. The different timescale is caused by the fact that depression is due to uncorrelated spikes originated by random noise, while pattern learning is linked to the density of patterns 1 or 2 and their related input frequency.

## Discussion

The proposed synapse circuit allows for asynchronous transmission and plasticity controlled by the spiking delay between the pre- and post-synaptic neurons. The synapse circuit adheres to the conventional organization of the neural network, where integrate-and-fire neurons serve as both input and output of the communication and plasticity. In particular, the BE terminal, being connected to the virtual ground input of the neuron, serves as reference ground for the synapse circuit, while pulses of arbitrary voltage are applied to the other 3 terminals, namely TE, CG and FG. This is different from previous approaches, where the pre-synaptic pulse (spike) and the post-synaptic pulse (fire) where applied to the TE and BE, respectively, of the resistive synapse (Yu et al., 2011; Indiveri et al., 2013). It is also different from other approaches employing 1T1R structures, where STDP relied on a dynamic V_T_ behavior of the transistor, achieved through nanoparticle-containing gate dielectric (Subramaniam et al., 2013). In fact, only standard transistor CMOS transistor are needed in the 2T1R synapse in this work.

The transistors in the 2T1R structure are functional in achieving 2 necessary behaviors of the synapse array, namely STDP and communication. On the one hand, the FG transistor allows for a spike timing comparison between two pulses, namely the TE pulse from the pre-synaptic neuron and the FG pulse from the post-synaptic neuron (Ambrogio et al., 2013). Therefore, the FG transistor is functional to plasticity. On the other hand, the CG transistor allows for enabling communication from pre-synaptic neuron to post-synaptic neuron in the neural network. If there was no CG transistor, the TE pulse might affect the weight of the synapse even without any fire from the post-synaptic neuron. Note in fact that the CG voltage is high only during the initial part of the TE pulse, at relatively low voltage. Therefore, this transistor is functional to communication, while protecting the memristor from the rather large TE voltage used for plasticity.

In addition, transistors allow to limit the current flowing in the memristive switch during the set transition, thus preventing uncontrolled switching and even irreversible breakdown of the device. These latter events may result in excessive power consumption due to low resistance value in the synapse, and/or in the impossibility to reset the memristor because of excessive growth of the conductive channel. Current limitation can be achieved by biasing the transistor in the saturated regime at relatively low gate voltage, which ensures that the maximum current after set transition is limited. Finally, the transistor serves as selector in the synapse array of Figure 9, which otherwise would be plagued by significant sneak-path currents (Baek et al., 2005). Note that other types of selectors would allow better scalability of the array, e.g., p-n diodes (Baek et al., 2005), or threshold switch devices (Cha et al., 2013), thanks to the 2-terminal structure. However, 2 terminals would not be sufficient for the local comparison of spike timing which is needed for synapse plasticity control.

**Figure 12:**
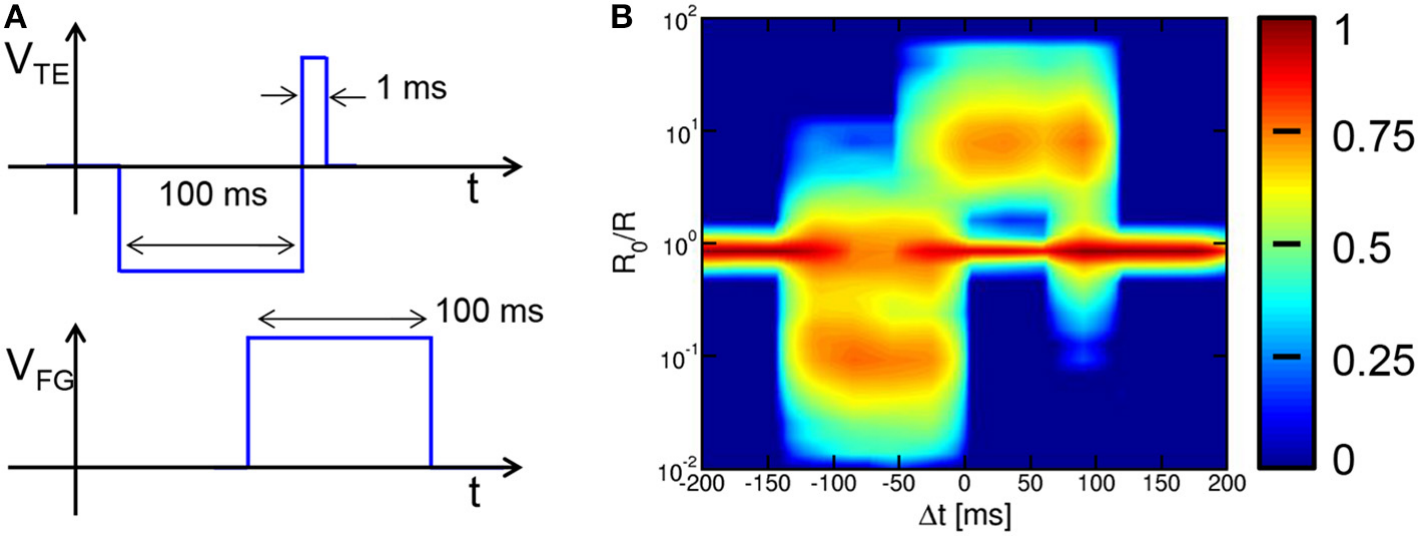
**Square-pulse STDP**. The use of square pulses for V_TE_ and V_FG_ **(A)** allows to achieve STDP with square characteristics suitable to learning and recognition **(B)**, while requiring a simple circuit for pulse generation.

It has been pointed out that the necessity to generate dedicated waveforms within the neuron circuit might lead to an excessive circuit overhead, thus conflicting with the need for very large scale arrays with high synaptic densities (Kornijcuk et al., 2014). Note however that the generator of the spike belongs to the neuron circuit, thus a complex waveform should not affect the density of synapses. Also, note that the waveforms in Figures 1, 2 have been designed to achieve a bio-realistic STDP as shown in Figure 4. Other waveforms and STDP characteristics can be used with no impact on the pattern recognition capability, while strongly alleviating the burden on the neuron circuit. This is demonstrated in Figure 12, showing the square waveforms for V_TE_ and V_FG_ (a) and the corresponding statistical STDP characteristic (b) obtained from 7.5 × 10^4^ random spikes. Note that the STDP characteristics reflects the simple shape of the spike and fire pulses, while we demonstrated that the pattern learning behavior is not affected. This further demonstrates the strength of the STDP process and the flexibility of our 2T1R circuit in realizing LTP and LTD with a variety of spike shapes. Note that pulse widths of the neuron spikes in the range of 100 ms, which are needed to achieve real-time bio-compatible neuromorphic behavior (Indiveri et al., 2011), do not necessarily require large capacitors. In fact, time responses in the 100 ms range are straightforwardly achieved in neuromorphic circuits through relatively small capacitances (e.g., 1 pF) charged/discharged by extremely low current in MOS transistors biased in the subthreshold regime (Mitra et al., 2009).

Low-power operation is a fundamental property of neuromorphic circuits. The energy consumption of our 2T1R synapse for communication can be estimated to about 150 nJ from the voltage waveform in Figure 1B assuming *I* = 50 μA. Assuming an average spike frequency of 1 Hz, the power consumption for communication should be around 150 nW. This value can be reduced by decreasing the pulse-width of the V_CG_ pulse and the current during communication. On the other hand, the energy consumption is slightly larger due to the larger voltage and current needed for resistive switching. For instance, the LTP energy is around 400 nJ for a current of 170 μA and a V_TE_ of 2.4 V in correspondence of the positive peak. However, since the LTP frequency is expected to be smaller than the spiking frequency, the power consumption for LTP might be in the same range as the communication power. Similar to the communication case, LTP power can be reduced by properly decreasing the current (e.g., by up to a factor 10) and the pulse width (up to a factor 10^3^). This allows for memristive-based synapses with relatively low power consumption.

Other switching concepts might be used in alternative to oxide memristors, e.g., spin-transfer-torque (STT) elements (Locatelli et al., 2014) or phase change memory (PCM) elements (Kuzum et al., 2012; Eryilmaz et al., 2014). However, oxide memristors allows for a smaller power consumption since the switching channel area can be controlled through the transistor current during the set transition, whereas the switching current is controlled by the lithography-defined area of the device in both STT and PCM devices, which thus can hardly be reduced below 50 μA (Ielmini and Lacaita, 2011; Kim et al., 2011).

The use of a HfO_2_ memristor allows for CMOS compatible process in the back-end, however other metal oxides can be used in principle for the active switching layer, such as TaO_x_ (Lee et al., 2011). A careful material engineering is needed to identify the best material properties for synaptic functionality, including, e.g., controllability of the synapse weight, stochastic switching and low power operation.

### Conflict of interest statement

The authors declare that the research was conducted in the absence of any commercial or financial relationships that could be construed as a potential conflict of interest.

## References

[B1] Abbott L. F. Nelson S. B. (2000). Synaptic plasticity: taming the beast. Nat. Neurosci. 3(Suppl), 1178–1183. 10.1038/81453 11127835

[B2] Ambrogio S. Balatti S. Cubeta A. Calderoni A. Ramaswamy N. Ielmini D. (2014a). Statistical fluctuations in HfO_x_ resistive-switching memory (RRAM): Part I – Set/Reset variability. IEEE Trans. Electron Devices 61, 2912–2919 10.1109/TED.2014.2330200

[B3] Ambrogio S. Balatti S. Gilmer D. C. Ielmini D. (2014b). Analytical modeling of oxide-based bipolar resistive memories and complementary resistive switches. IEEE Trans. Electron Devices 61, 2378–2386 10.1109/TED.2014.2325531

[B4] Ambrogio S. Balatti S. Nardi F. Facchinetti S. Ielmini D. (2013). Spike-timing dependent plasticity in a transistor-selected resistive switching memory. Nanotechnology 24:384012. 10.1088/0957-4484/24/38/384012 23999495

[B5] Baek I. G. Kim D. C. Lee M. J. Kim H.-J. Yim E. K. Lee M. S. (2005). Multi-layer cross-point binary oxide resistive memory (OxRRAM) for Post-NAND storage application. IEDM Tech. Dig. 750–753 10.1109/IEDM.2005

[B6] Bi G.-Q. Poo M.-M. (1998). Synaptic modifications in cultured hippocampal neurons: dependence on spike timing, synaptic strength, and postsynaptic cell type. J. Neurosci. 18, 10464–10472. 9852584 10.1523/JNEUROSCI.18-24-10464.1998 PMC6793365

[B7] Bichler O. Suri M. Querlioz D. Vuillaume D. DeSalvo B. Gamrat C. (2012). Visual pattern extraction using energy-efficient 2-PCM synapse neuromorphic architecture. IEEE Trans. Electron Devices 59, 2206–2214 10.1109/TED.2012.2197951

[B8] Bichler O. Zhao W. Alibart F. Pleutin S. Vuillaume D. Gamrat C. (2010). Functional model of a nanoparticle organic memory transistor for use as a spiking synapse. IEEE Trans. Electron Devices 57, 3115–3122 10.1109/TED.2010.2065951

[B9] Calderoni A. Sills S. Ramaswamy N. (2014). Performance comparison of O-based and Cu-based ReRAM for high-density applications, in International Memory Workshop (Taipei), 1–4.

[B10] Caporale N. Dan Y. (2008). Spike timing-dependent plasticity: a hebbian learning rule. Annu. Rev. Neurosci. 31, 25–46. 10.1146/annurev.neuro.31.060407.125639 18275283

[B11] Cha E. Woo J. Lee D. Lee S. Song J. Koo Y. (2013). Nanoscale (~10nm) 3D vertical ReRAM and NbO_2_ threshold selector with TiN electrode. IEDM Tech. Dig. 268–271 10.1109/IEDM.2013.6724602

[B12] Diorio C. J. Hasler P. E. Mead C. A. Minch B. A. (1996). A single-transistor silicon synapse. IEEE Trans. Electron Devices 43, 1972–1980 10.1109/16.543035

[B13] Eryilmaz S. B. Kuzum D. Jeyasingh R. Kim S. BrightSky M. Lam C.. (2014). Brain-like associative learning using a nanoscale non-volatile phase change synaptic device array. Front. Neurosci. 8:205. 10.3389/fnins.2014.00205 25100936 PMC4106403

[B14] Ielmini D. (2011). Modeling the universal set/reset characteristics of bipolar RRAM by field- and temperature-driven filament growth. IEEE Trans. Electron Devices 58, 4309–4317 10.1109/TED.2011.2167513

[B15] Ielmini D. Lacaita A. L. (2011). Phase change materials in non-volatile storage. Mater. Today 14, 600–607 10.1016/S1369-7021(11)70301-7

[B16] Indiveri G. Chicca E. Douglas R. (2006). A VLSI array of low-power spiking neurons and bistable synapses with spike-timing dependent plasticity. IEEE Trans. Neural Netw. 17, 211–221. 10.1109/TNN.2005.860850 16526488

[B17] Indiveri G. Linares-Barranco B. Hamilton T. J. van Schaik A. Etienne-Cummings R. Delbruck T.. (2011). Neuromorphic silicon neuron circuits. Front. Neurosci. 5:73. 10.3389/fnins.2011.00073 21747754 PMC3130465

[B18] Indiveri G. Linares-Barranco B. Legenstein R. Deligeorgis G. Prodromakis T. (2013). Integration of nanoscale memristor synapses in neuromorphic computing architectures. Nanotechnology 24:384010. 10.1088/0957-4484/24/38/384010 23999381

[B19] Jo S. H. Chang T. Ebong I. Bhadviya B. B. Mazumder P. Lu W. (2010). Nanoscale memristor device as synapse in neuromorphic systems. Nano Lett. 10, 1297–1301. 10.1021/nl904092h 20192230

[B20] Kim W. Jeong J. H. Kim Y. Lim W. C. Kim J. H. Park J. H. (2011). Extended scalability of perpendicular STT-MRAM towards sub-20nm MTJ node. IEDM Tech. Dig. 531–534 10.1109/IEDM.2011.6131602

[B21] Kinoshita K. Tsunoda K. Sato Y. Noshiro H. Yagaki S. Aoki M. (2008). Reduction in the reset current in a resistive random access memory consisting of NiO_x_ brought about by reducing a parasitic capacitance. Appl. Phys. Lett. 93, 033506 10.1063/1.2959065

[B22] Kornijcuk V. Kavehei O. Lim H. Seok J. Y. Kim S. K. Kim I.. (2014). Multiprotocol-induced plasticity in artificial synapses. Nanoscale 6, 15151–15160. 10.1039/C4NR03405H 25373422

[B23] Kuzum D. Jeyasingh R. G. D. Lee B. Wong H.-S. P. (2012). Nanoelectronic programmable synapses based on phase change materials for brain-inspired computing. Nano Lett. 12, 2179–2186. 10.1021/nl201040y 21668029

[B24] Lee H. Y. Chen P. S. Wu T. Y. Chen Y. S. Wang C. C. Tzeng P. J. (2008). Low power and high speed bipolar switching with a thin reactive Ti buffer layer in robust HfO_2_ based RRAM. IEDM Tech. Dig. 297–300 10.1109/IEDM.2008.4796677

[B25] Lee M.-J. Lee C. B. Lee D. Lee S. R. Chang M. Hur J. H.. (2011). A fast, high-endurance and scalable non-volatile memory device made from asymmetric Ta_2_O_5-x_/TaO_2-x_ bilayer structures. Nat. Mater. 10, 625–630. 10.1038/nmat3070 21743450

[B26] Likharev K. K. Mayr A. Muckra I. Türel Ö. (2003). CrossNets – high-performance neuromorphic architectures for CMOL circuits. Ann. N.Y. Acad. Sci. 1006, 146–163. 10.1196/annals.1292.010 14976016

[B27] Locatelli N. Cros V. Grollier J. (2014). Spin-torque building blocks. Nat. Mater. 13, 11–20. 10.1038/nmat3823 24343514

[B28] Mitra S. Fusi S. Indiveri G. (2009). Real-time classification of complex patterns using spike-based learning in neuromorphic VLSI. IEEE Trans. Biomed. Cir. Syst. 3, 32–43. 10.1109/TBCAS.2008.2005781 23853161

[B29] Nardi F. Larentis S. Balatti S. Gilmer D. C. Ielmini D. (2012). Resistive switching by voltage-driven ion migration in bipolar RRAM – Part I: experimental study. IEEE Trans. Electron Devices 59, 2461–2467 10.1109/TED.2012.2202319

[B30] Nishiyama M. Hong K. Mikoshiba K. Poo M.-M. Kato K. (2000). Calcium stores regulate the polarity and input specificity of synaptic modification. Nature 408, 584–588. 10.1038/35022604 11117745

[B31] Ohno T. Hasegawa T. Tsuruoka T. Terabe K. Gimzewski J. K. Aono M. (2011). Short-term plasticity and long-term potentiation mimicked in single inorganic synapses. Nat. Mater. 10, 591–595. 10.1038/nmat3054 21706012

[B32] Park S. Kim H. Choo M. Noh J. Sheri A. Jung S. (2012). RRAM-based synapse for neuromorphic system with pattern recognition function, in Electron Devices Meeting (IEDM), 2012 IEEE International (San Francisco, CA). 10.1109/IEDM.2012.6479016

[B33] Rajendran B. Liu Y. Seo J.-S. Gopalakrishnan K. Chang L. Friedman D. J. (2013). Specifications of nanoscale devices and circuits for neuromorphic computational systems. IEEE Trans. Electron Devices 60, 246–253 10.1109/TED.2012.2227969

[B34] Seo K. Kim I. Jung S. Jo M. Park S. Park J.. (2011). Analog memory and spike-timing-dependent plasticity characteristics of a nanoscale titanium oxide bilayer resistive switching device. Nanotechnology 22:254023. 10.1088/0957-4484/22/25/254023 21572200

[B35] Serrano-Gotarredona T. Masquelier T. Prodromakis T. Indiveri G. Linares-Barranco B. (2013). STDP and STDP variations with memristors for spiking neuromorphic learning systems. Front. Neurosci. 7:2. 10.3389/fnins.2013.00002 23423540 PMC3575074

[B36] Snider G. S. (2008). Spike-timing-dependent learning in memristive nanodevices, in IEEE/ACM International Symposium on Nanoscale Architectures, NANOARCH (Anaheim), 85–92.

[B37] Subramaniam A. Cantley K. D. Bersuker G. Gilmer D. C. Vogel E. M. (2013). Spike-timing-dependent plasticity using biologically realistic action potentials and low-temperature materials. IEEE Trans. Nanotechnol. 12, 450–454 10.1109/TNANO.2013.2256366

[B38] Suri M. Querlioz D. Bichler O. Palma G. Vianello E. Vuillaume D. (2013). Bio-inspired stochastic computing using binary CBRAM synapses. IEEE Trans. Electron Devices 60, 2402–2409 10.1109/TED.2013.2263000

[B39] Wittenberg G. M. Wang S. S.-H. (2006). Malleability of spike-timing-dependent plasticity at the CA3–CA1 synapse. J. Neurosci. 26, 6610–6617. 10.1523/JNEUROSCI.5388-05.2006 16775149 PMC6674029

[B40] Wong H.-S. P. Lee H.-Y. Yu S. Chen Y.-S. Wu Y. Chen P.-S. (2012). Metal–Oxide RRAM. Proc. IEEE 100, 1951–1970 10.1109/JPROC.2012.2190369

[B41] Wright C. D. Liu Y. Kohary K. I. Aziz M. M. Hicken R. J. (2011). Arithmetic and Biologically-inspired computing using phase-change materials. Adv. Mater. 23, 3408–3413. 10.1002/adma.201101060 21695736 PMC3715110

[B42] Yu S. Gao B. Fang Z. Yu H. Kang J. Wong H.-S. P. (2013). A low energy oxide-based electronic synaptic device for neuromorphic visual systems with tolerance to device variation. Adv. Mater. 25, 1774–1779. 10.1002/adma.201203680 23355110

[B43] Yu S. Wu Y. Jeyasingh R. Kuzum D. Wong H.-S. P. (2011). An electronic synapse device based on metal oxide resistive switching memory for neuromorphic computation. IEEE Trans. Electron Devices 58, 2729–2737 10.1109/TED.2011.2147791

[B44] Zamarreño-Ramos C. Camuñas-Mesa L. A. Pérez-Carrasco J. A. Masquelier T. Serrano-Gotarredona T. Linares-Barranco B. (2011). On spike-timing-dependent-plasticity, memristive devices, and building a self-learning visual cortex. Front. Neurosci. 5:26. 10.3389/fnins.2011.00026 21442012 PMC3062969

